# Inaccuracy of a non-invasive estimation of pulmonary vascular resistance assessed by cardiovascular magnetic resonance in heart failure patients

**DOI:** 10.1038/s41598-021-95897-5

**Published:** 2021-08-16

**Authors:** Eva Rumiz, Andrés Cubillos, Juan Vicente Vilar, Pilar García, Alberto Berenguer, Lorenzo Fácila, Ernesto Valero, Verónica Vidal, Salvador Morell, Julio Nuñez

**Affiliations:** 1 Cardiology Department, Consorcio Hospital General Universitario de Valencia, Av. Tres Cruces Nº 2, 46014 Valencia, Spain; 2 Cardiology Department, Hospital QuironSalud Valencia, Valencia, Spain; 3 Cardiovascular Unit, Ascires Biomedical Group, Valencia, Spain; 4 Cardiology Department, Hospital Clínico Universitario de Valencia, INCLIVA, Valencia, Spain; 5 CIBER Cardiovascular, Madrid, Spain

**Keywords:** Cardiology, Interventional cardiology

## Abstract

Pulmonary vascular resistance (PVR) is a marker of pulmonary vascular remodeling. A non-invasive model assessed by cardiovascular magnetic resonance (CMR) has been proposed to estimate PVR. However, its accuracy has not yet been evaluated in patients with heart failure. We prospectively included 108 patients admitted with acute heart failure (AHF), in whom a right heart catheterization (RHC) and CMR were performed at the same day. PVR was estimated by CMR applying the model: PVR = 19.38 − [4.62 × Ln pulmonary artery average velocity (in cm/s)] − [0.08 × right ventricle ejection fraction (in %)], and by RHC using standard formulae. The median age of the cohort was 67 years (interquartile range 58–73), and 34% were females. The median of PVR assessed by RHC and CMR were 2.2 WU (1.5–4) and 5 WU (3.4–7), respectively. We found a weak correlation between invasive PVR and PVR assessed by CMR (Spearman r = 0.21, p = 0.02). The area under the ROC curve for PVR assessed by CMR to detect PVR ≥ 3 WU was 0.57, 95% confidence interval (CI): 0.47–0.68. In patients with AHF, the non-invasive estimation of PVR using CMR shows poor accuracy, as well as a limited capacity to discriminate increased PVR values.

## Introduction

Pulmonary hypertension (PH) is a common finding in patients with heart failure (HF), regardless of left ventricular ejection fraction (LVEF) status^1^. The development of PH overshadows the prognosis of these patients, leading to right ventricular dysfunction and an increased risk of mortality^2,3^. The backward transmission of elevated left-sided filling pressures into the pulmonary circulation is the hallmark of isolated post-capillary PH (IpcPH). However, in many cases venous congestion is followed by vasoconstriction and arterial remodeling, leading to an increased pulmonary vascular resistance (PVR), superimposing a pre-capillary component, which has been defined as combined PH (Cpc-PH). In this regard, PVR has become a useful marker of pulmonary arterial remodeling with crucial diagnostic and prognostic value, involving important therapeutic implications^4^.

Currently, the gold-standard technique for quantifying PVR is invasively, through right heart catheterization (RHC). Cardiovascular magnetic resonance (CMR), which also provides us with an excellent study of the right ventricle (RV) and pulmonary circulation, has recently been postulated as a useful technique for estimating non-invasively the PVR^5^. García-Alvarez et al. showed a strong correlation with invasive PVR and PVR derived from a model based on right ventricular ejection fraction (RVEF) and the natural log of average pulmonary arterial velocity in an heterogenous PH population^6^. However, its performance in a specific and larger group of PH due to left heart disease (PH-LHD) is still unknown. Accordingly, we sought to evaluate the diagnostic accuracy of a noninvasive CMR model for the estimation of PVR in a cohort of patients admitted with acute HF (AHF).

## Methods

### Study population

We conducted a prospective, observational study that included 154 consecutive patients admitted with AHF, between February 2015 and May 2019, who were referred to the catheterization laboratory of our hospital in order to perform a RHC and in whom a CMR was realized during hospitalization. Both techniques were performed after initiating optimal medical treatment and when hemodynamic stability and congestion improvement were achieved, without inotropic agents requirements and always to the discretion of the attending physician.

AHF was defined according to European Society of Cardiology guidelines as rapid onset or worsening of symptoms and/or signs of HF^7^. Both, a first occurrence and an acute decompensation of chronic HF were included. Patients with congenital heart disease (n = 12) and those with organic valvular disease (n = 14) were excluded from this study. In addition, we excluded patients in which RHC and CMR were performed with an interval of time of more than 6 h of difference between both (n = 20) in order to avoid discrepancies in hemodynamic status. A total of 108 patients were finally enrolled.

In order to evaluate differences in the diagnostic accuracy, subgroup analysis was performed classifying our study population according to (1) the median of LVEF, and (2) the presence of preserved (≥ 45%) or reduced (< 45%) RVEF.

All participants provided written informed consent and the local ethics committee (Comité ético de investigación clínica del Consorcio Hospital General Universitario de Valencia) approved the study. The study protocol conformed to the ethical guidelines of the 1975 Declaration of Helsinki (revised in 1983) as reflected by an a priori approval by the institution´s human research committee.

### Cardiovascular magnetic resonance

CMR was performed with a 1.5 T Siemens system (Magnetom Sonata, Siemens; Erlangen, Germany). For cine imaging, breath-holding ECG-gated steady-state free precession (SSFP) sequences were used as normally to acquire long and short axis slices, and hence evaluate ventricular volumes and function. 2D flow imaging was performed perpendicular to the pulmonary artery (PA) trunk with a velocity encoded phase-contrast sequence using an upper-velocity limit of 150 cm/s (or the minimum velocity without signal aliasing). Two double-oblique orthogonal views oriented along the main PA were acquired with SSFP cine sequence and used as the reference to prescribe the plane perpendicular to the PA trunk for the acquisition of phase-contrast images. These parameters were applied as usually: field of view 300 × 400 mm, acquisition matrix 256/192, slice thickness 6 mm, time/echo time 5.9–7.5/3.1–6.5 ms, in-plane resolution 1.5–3 mm, 30 reconstructed cardiac phases, and temporal resolution 55–105 ms. 2D flow CMR acquisitions were performed during free breathing and retrospective ECG-gating was used^8^.

Images were analyzed by a single expert cardiologist in cardiac imaging using specific software (Argus, Siemens, Erlangen, Germany). Short axis slices were used to calculate ejection fractions and ventricular volumes using Simpson's method. PA cross-section was outlined in each cardiac phase to estimate PA area and flow, and calculate peak and average velocities during the complete cardiac cycle, minimum and maximum areas, and PA net forward volume. Ventricular volumes, ejection fractions, and PA area were adjusted to body surface area^9^.

PVR were calculated using the following model proposed by García-Alvarez et al.^6^: PVR (WU) = 19.38 − [4.62 × ln PA average velocity (cm/s)] − [0.08 × RVEF (%)].

### Right heart catheterization

RHC was performed using a 7F Swan–Ganz catheter via a femoral or brachial vein approach after optimizing diuretic treatment, and heart rate control in patients with atrial fibrillation. The following hemodynamic measurements were always recorded during end-expiration: mean right atrial pressure; systolic, diastolic and mean pulmonary artery pressure (mPAP); systolic, mean, and diastolic right ventricle pressure and mean pulmonary arterial wedge pressure (PAWP). PAWP was measured 130–160 ms after the onset of QRS and before the v-wave in patients with atrial fibrillation. Cardiac output was determined as the mean of three measurements using the thermodilution method. Fick method was used when a significant tricuspid regurgitation was present. Transpulmonary gradient (TPG) was calculated by subtracting PAWP from mean PAP, and PVR as TPG divided by RV cardiac output and expressed in WU. The diagnosis of PH-LHD was established when mPAP was > 20 mmHg and PAWP > 15 mmHg, according to recent PH diagnostic criteria^10^. Increased PVR was defined as ≥ 3 WU.

### Statistical analysis

Continuous variables were expressed as median with interquartile range. Discrete variables were summarized as frequency and percentages.

The correlations between RHC (PVR_RHC_) with CMR (PVR_CMR_) and different other CMR parameters (RVEF, RV end-diastolic and end-systolic volume, PA maximal and minima area, PA average and peak velocity, PA forward volume and LVEF) were evaluated by Spearman correlation analysis. Bland–Altman analysis was performed to assess the degree of agreement between PVR_CMR_ and PVR_RHC_. The bias, standard deviation, and the 95% limits of agreement were reported. Receiver operating characteristics (ROC) curves were constructed to determine the diagnostic accuracy of non-invasive model for the detection of increased values of PVR (≥ 3 WU). Area under the ROC curve ≤ 0.5 indicates no value. The closer the area is to 1.0, the greater the diagnostic utility and significance of the test. A p-value < 0.05 was considered statistically significant. STATA 14.1 software was used to perform and display the statistical analysis (StataCorp. 2014. Stata Statistical Software: Release 14.1. College Station, TX, USA).

## Results

Demographic characteristics, CMR indices, and RHC measurements of our study cohort are summarized in Table 1. The median age was 67 years (interquartile range 58–73), 34% were females and 27% showed ischemic etiology. Ninety four patients (87%) had reduced LVEF, and the median of N-terminal pro-B-type natriuretic peptide (NT-proBNP) was 3851 pg/ml (interquartile range 576–5406). RHC and CMR were performed during index admission at the same day with a median time of 4 days (3–6) since patient admission.

**Table 1:** Clinical, right heart catheterization and cardiovascular magnetic resonance variables.

Variable	
**Demographics**	
Age, y	67 (58–73)
Female, n (%)	34 (31.5)
Hypertension, n (%)	69 (64)
Diabetes mellitus, n (%)	37 (40)
Dyslipidemia, n (%)	49 (45.4)
Former smoker, n (%)	29 (28)
COPD, n (%)	17 (16)
CKD^a^, n (%)	19 (17.6)
Coronary artery disease, n (%)	29 (27)
Atrial fibrillation, n (%)	35 (32.4)
**Laboratory**	
Serum creatinine, mg/dl	0.9 (0.7–1)
NT-proBNP, pg/ml	3851 (576–5406)
**RHC measurements**	
Right atrial pressure, mmHg	10 (7–14)
Systolic PAP, mmHg	50 (35–60)
Mean PAP, mmHg	30 (21–38)
CO (l/min)	3.7 (3–5)
PAWP, mmHg	19 (14–25)
TPG, mmHg	10 (6.2–15)
PVR, Wood units	2.2 (1.5–4)
PVR ≥ 3 Wood units, n (%)	45 (42)
**CMR parameters**	
LVEDV, ml/m^2^	110 (83–137)
LVESV, ml/m^2^	71 (48–108)
LVEDD, mm	57 (53–64.5)
LVESD, mm	49 (41–57)
LVEF, %	30 (20–40)
RVEDV, ml/m^2^	69 (50–99)
RVESV, ml/m^2^	38 (24–63)
RVEDD, mm	36 (27–41)
RVEF, %	43 (30–53)
Maximal PA area, cm/m^2^	4.6 (3.2–5.3)
Minimal PA area, cm/m^2^	3.4 (2.6–4.4)
PA peak velocity, cm/s	56 (43–77)
PA average velocity, cm/s	8.6 (5.5–12.8)
PA net forward volume, ml	30 (22.7–38)
PVR, Wood units	5 (3.4–7)
PVR, ≥ 3, Wood units	71 (65.7)

Data are given as n (%), or median (interquartile range). *CMR* cardiovascular magnetic resonance, *CKD* chronic kidney disease, *CO* cardiac output, *COPD* chronic obstructive pulmonary disease, *LVEDD* left ventricular end-diastolic diameter, *LVEDV* left ventricular end-diastolic volume, *LVEF* left ventricular ejection fraction, *LVESD* left ventricular end-systolic diameter, *LVESV* left ventricular end-systolic volume, *NT-proBNP* N-terminal pro-B-type natriuretic peptide, *PA* pulmonary artery, *PAWP* pulmonary arterial wedge pressure, *PAP* pulmonary artery pressure, *PVR* pulmonary vascular resistance, *RHC* right heart catheterization, *RVEDD* right ventricular end-diastolic diameter, *RVEDV* right ventricular end-diastolic volume, *RVEF* right ventricular ejection fraction, *RVESV* right ventricular end-systolic volume, *RVP* right ventricular pressure, *TPG* transpulmonary gradient.

^a^Estimated glomerular filtration rate < 60 ml/min/1.73 m^2^ by Cockcroft–Gault equation.

### Right heart catheterization

The median (interquartile range) of mPAP and PAWP was 30 mmHg (21–38) and 19 mmHg (14–25), respectively. The median (interquartile range) of PVR_RHC_ was 2.2 UW (1.5–4), and for right ventricle cardiac output was 3.7 l/min (3–5), using the thermodilution technique in 101 (93.5%) patients. The number of patients with PH-LHD was 84 patients (77.8%), and 45 patients (42%) exhibited CpcPH at diagnosis.

### Cardiovascular magnetic resonance

The median (interquartile range) of LVEF and RVEF were 30% (20–41) and 43% (30–53), respectively. Fourteen patients (13%) showed preserved LVEF (≥ 50%), and 59 patients (55%) presented RVEF < 45%. The median (interquartile range) of PVR_CMR_ was 5 WU (3.4–7) and 71 patients (65.7%) exhibited PVR values ≥ 3 WU.

### Correlation between cardiac magnetic resonance and right heart catheterization

**Figure 1:**
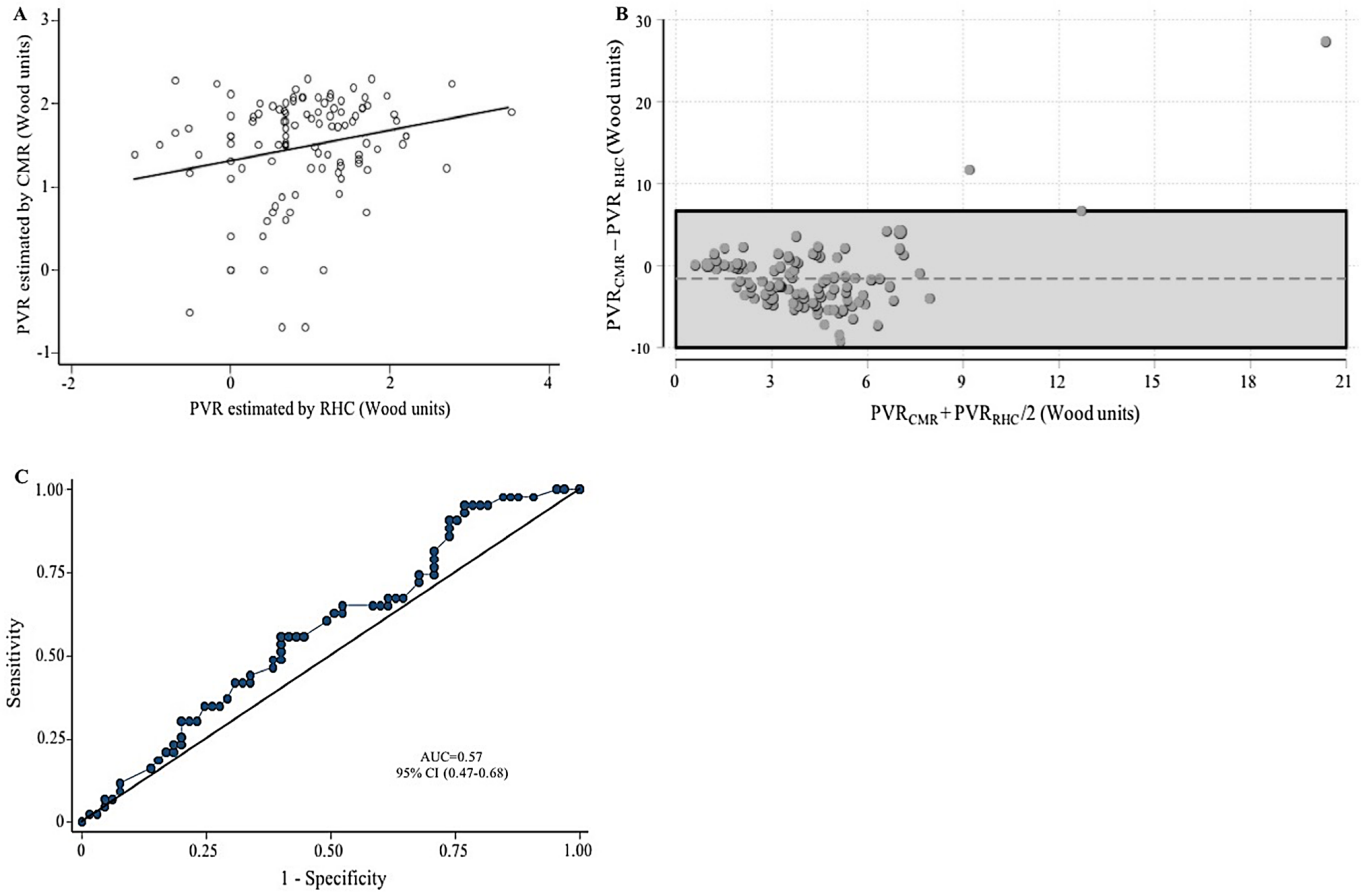
Accuracy of non-invasive cardiovascular magnetic resonance model. (**A**) Correlation between pulmonary vascular resistance quantified by right heart catheterization and pulmonary vascular resistance assessed by cardiovascular magnetic resonance. (**B**) Bland–Altman analysis. (**C**) Area under the receiver operator characteristics curve with 95% confidence interval for the detection of increased pulmonary vascular resistances (≥ 3 WU) using the cardiovascular magnetic resonance model. *AUC* area under the receiver operator characteristics curve, *CI* confidence interval, *CMR* cardiovascular magnetic resonance, *PVR* pulmonary vascular resistance, *PVR*_*RHC*_ pulmonary vascular resistance assessed by right heart catheterization, *PVR*_*CMR*_ pulmonary vascular resistance estimated by cardiovascular magnetic resonance, *RHC* right heart catheterization.

Our study population showed a weak positive correlation between invasive PVR and PVR assessed by CMR (Spearman r = 0.21, p = 0.02) as is shown in Fig. 1A. Bland–Altman's analysis revealed that the mean bias was − 1.7, and the 95% limits of agreement ranged from − 10.02 to 6.6 WU (Fig. 1B). The area under the ROC curve for PVR_CMR_ to detect PVR ≥ 3 WU was 0.57, 95% confidence interval (CI): 0.47–0.68, (Fig. 1C).

Several correlations between CMR parameters and PVR_RHC_ are summarized in Table 2. We found a weak negative correlation with PA average velocity (Spearman r = − 0.21, p = 0.04) and PA peak velocity (Spearman r = − 0.27, p = 0.005), as well as PA forward net volume (Spearman r = − 0.26, p = 0.009). We did not find statistical correlation between PVR_RHC_ and RVEF (Spearman r = − 0.12, p = 0.20).

**Table 2:** Spearman correlation coefficients between pulmonary vascular resistance and cardiovascular magnetic resonance parameters.

Variable (N = 108)	r	p value
RV ejection fraction	− 0.12	0.20
RV end-diastolic volume	0.13	0.23
RV end-systolic volume	0.15	0.16
PA maximal area	0.008	0.93
PA minimal area	0.008	0.93
PA average velocity	− 0.21	0.04
PA peak velocity	− 0.27	0.005
PA forward net volume	− 0.26	0.009
LV ejection fraction	− 0.07	0.45

*LV* left ventricle, *PA* pulmonary artery, *RV* right ventricle.

### Subgroup analysis

**Figure 2:**
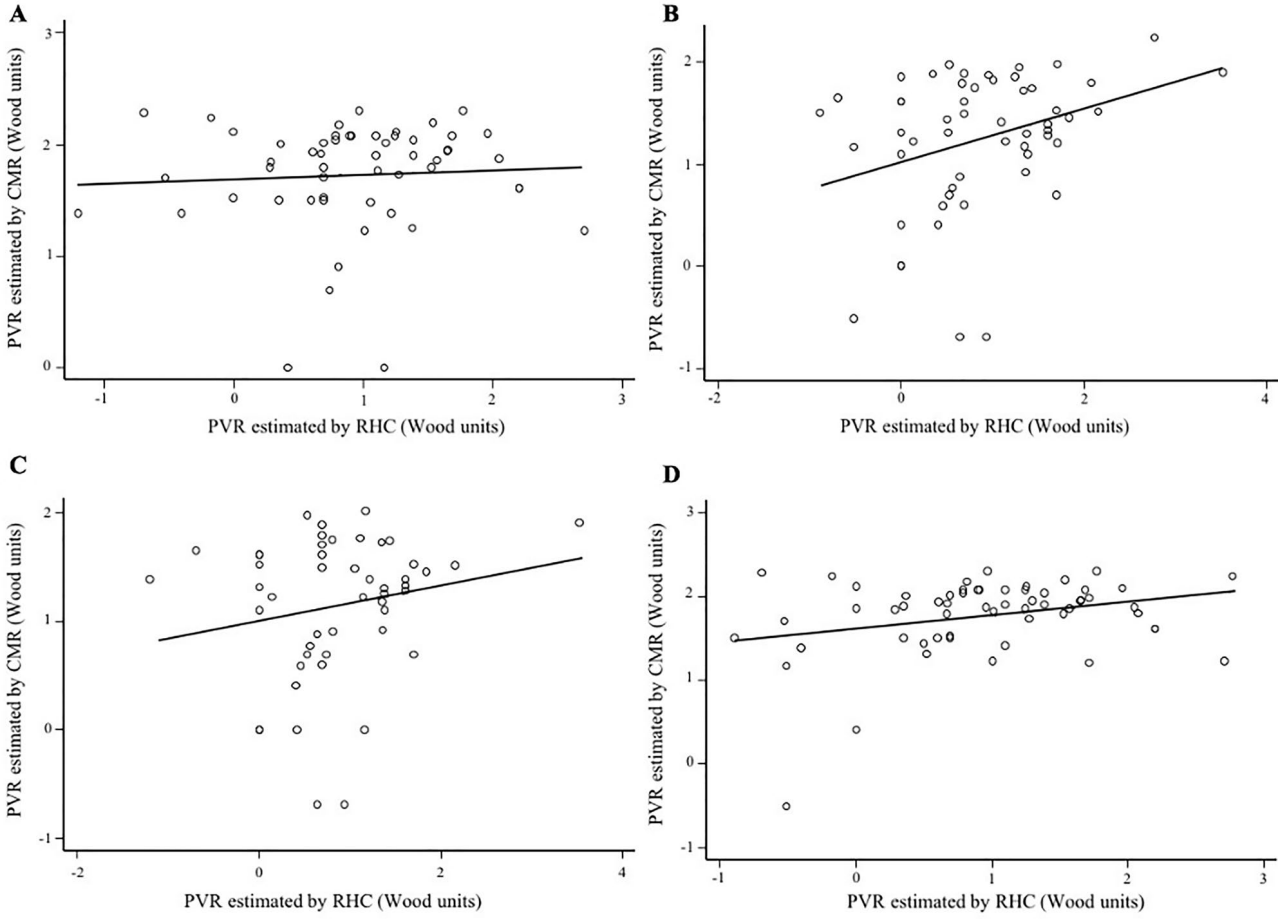
Subgroup correlations between pulmonary vascular resistance quantified by right heart catheterization and pulmonary vascular resistance assessed by cardiovascular magnetic resonance. (**A**) Left ventricular ejection fraction less than 30%. (**B**) Left ventricular ejection fraction more or equal to 30%. (**C**) Depressed right ventricular ejection fraction. (**D**) Preserved right ventricular ejection fraction. *CMR* cardiovascular magnetic resonance, *PVR* pulmonary vascular resistance, *RHC* right heart catheterization.

**Figure 3:**
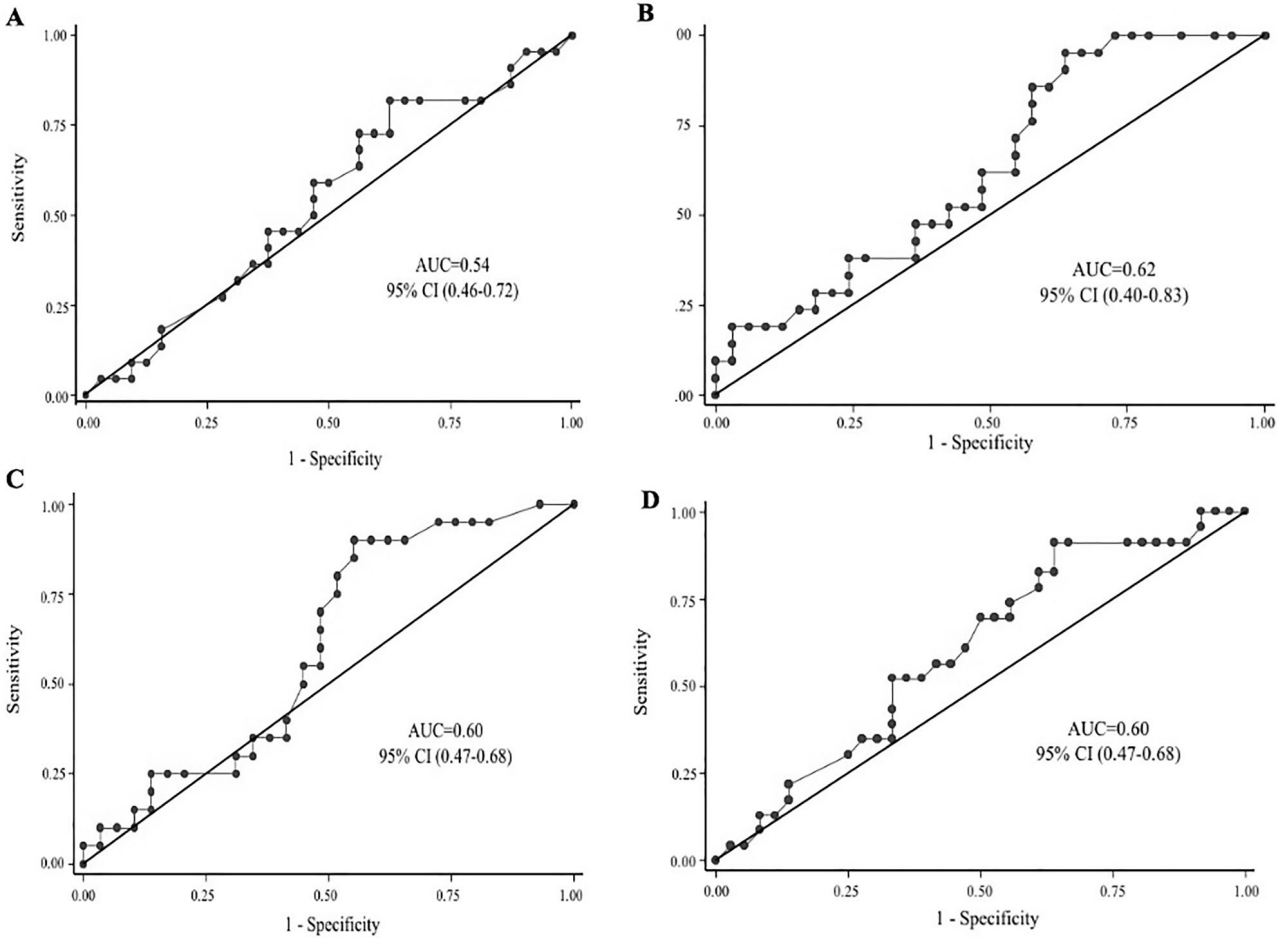
Subgroup analysis of area under the receiver operator characteristics curve with 95% confidence interval for the cardiovascular magnetic resonance model to detect increased pulmonary vascular resistance (≥ 3 WU). (**A**) Left ventricular ejection fraction less than 30%. (**B**) Left ventricular ejection fraction more or equal to 30%. (**C**) Depressed right ventricular ejection fraction. (**D**) Preserved right ventricular ejection fraction. *AUC* area under the receiver operator characteristics curve, *CI* confidence interval, *CMR* cardiovascular magnetic resonance, *PVR* pulmonary vascular resistance, *RHC* right heart catheterization.

When analyzing the correlation according to the median of LVEF (< 30% versus ≥ 30%), we observed no correlation between PVR_CMR_ and PVR_RHC_ in patients with LVEF < 30% (n = 54) (Spearman r = 0.11, p = 0.40), (Fig. 2A). On the contrary, patients with LVEF ≥ 30% (n = 54) showed positive agreement, although poor, between both methods (Spearman r = 0.29, p = 0.02), (Fig. 2B). Regarding RVEF status, we also found no correlation in both, preserved and reduced RVEF (Spearman r = 0.17, p = 0.22 and Spearman and r = 0.21, p = 0.09, respectively), (Fig. 2C,D). A low area under the ROC curve for PVR_CMR_ to detect PVR ≥ 3 WU, was observed in both, LVEF < 30% 0.54, 95% CI (0.46–0.72) and LVEF ≥ 30% 0.62, 95% CI (0.40–0.83), (Fig. 3A,B); as well as, for RVEF < 45% and RVEF ≥ 45%, that were 0.60, 95% CI (0.45–0.75) and 0.60, 95% CI (0.44–0.76), respectively (Fig. 3C,D).

## Discussion

To the best of our knowledge, this is the first study designed to evaluate the diagnostic accuracy of a non-invasive PVR method in a specific population of AHF patients. Our results suggest that the CMR method fails to estimate with accuracy PVR. Furthermore, the present findings confirm its lack of ability to discriminate with precision increased values of PVR in a specific group of HF patients.

The development of PH is a common complication in the progression of HF, regardless of LVEF^1^. PH-LHD is the most common form of PH, with a prevalence that ranges between 36–83%^11,12^. Currently, PVR is a well-established prognostic marker in HF patients^2^. Several studies suggest that HF patients with increased values of PVR, are associated with worse survival and an increase in HF hospitalizations^4,12^. In this sense, an accurate and reproductible non-invasive method capable of estimating PVR remains an unmet need in HF patients. Different studies have reported promising results with different models based in CMR imaging metrics, but all of them have several limitations, either due to a small sample size, retrospective cohorts, or because results were validated predominantly in pulmonary arterial hypertension (PAH) patients^13–18^.

García-Alvarez et al.^6^ developed a non-invasive model assessed by CMR based on RVEF and mean PA velocity to predict PVR. They analyzed a suspected PH cohort of 100 patients divided in a homogeneous derivation and validation cohort of 80 and 20 patients, respectively. It is important to highlight, that this study included different etiologies of PH, although PAH patients were the vast majority. Only 20 patients (20%) in the derivation cohort and 3 patients (15%) in the validation cohort exhibited PH-LHD. The overall population showed a median LVEF within normal ranges (58.7%), in contrast to our HF population that showed a severely depressed LVEF, with only fourteen patients (13%) displaying LVEF ≥ 50%. Nevertheless, the median RVEF was depressed, quite similar in both studies (43% versus 40.5%). Regarding on RHC measurements, the study population showed a mPAP of 39 mmHg, slightly superior when compared to our group, but with a low value of PAWP (9 mmHg) and right atrial pressure (7 mmHg), reflecting a clear prevalence of precapilar PH phenotypes. It is noteworthy, that these patients showed a great pulmonary vascular remodeling with median RVP_RHC_ of 4.7 WU and 66% exhibited PVR ≥ 3 WU. By contrast, our HF population had a median PVR below normal value and a less percentage of patients had PVR above normal value, maybe translating initial stages of PH-LHD or the hemodynamic pattern of different PH groups.

García-Alvarez et al. showed a strong correlation between the non-invasive model and PVR_RHC_ in their precapillary PH cohort (Spearman r = 0.84, p < 0.001). On Bland–Altman analysis the mean bias was − 0.54 ± 2.80 with wide limits of agreement − 6.02 to 4.94 WU. Our study, performed in a specific group of HF patients, observed by contrast a weak correlation with an important mean bias and broad range limits of agreement on Bland–Altman analysis, that translate an inadequate diagnostic accuracy in this specific HF population. In our subgroup analysis, where we searched differences in diagnostic accuracy according to the median of LVEF, we did neither find an important agreement between both methods. Similar results were observed when RVEF was evaluated.

The CMR-method was created using CMR variables which displayed the strongest correlation with PVR_RHC_. Pulmonary artery velocity and RVEF were the parameters that exhibited the strongest univariate correlation. In our current study, a scarce negative agreement was observed between PVR_RHC_ and average PA velocity. This negative correlation has just been widely reported in patients with PAH^19,20^, and has been postulated as a surrogate of PH. However, the evidence describing how PA average velocity decreases assessed by CMR in the specific group of PH-LHD patients is scarce. We hypothesized that the decline of PA velocity could differ in patients with PH-LHD, especially in early stages when no component of the PH is derived from abnormalities intrinsic to the pulmonary arterial bed. This factor can be related with the lack of agreement of this method. On the other hand, no correlation with RVEF was found. Therefore, we speculate that RV dysfunction in our HF population is mainly due to the presence of a cardiomyopathic RV and does not translate solely an end-stage uncoupling of the RV and its load. We should underline, that another potential factor that can contribute to the inaccuracy of the method in this specific population is the lack of any variable that reflects left chamber pressures, essential in the pathophysiology of PH-LHD and maybe imperative in a non-invasive PVR evaluation in HF patients.

García-Alvarez et al. also explored the capability to differentiate increased values of PVR, observing an excellent discrimination ability of the model in the study population, obtaining an area under the ROC curve of 0.96, 95% CI: 0.92–0.99, which was reassured in the validation cohort. Nevertheless, our results showed a lack of discrimination power of this method to detect elevated PVR in our HF cohort. It must be noted that the agreement was worse in patients with PVR values above the normal limit.

These results may translate that this non-invasive method should only be recommended for the evaluation of PVR in patients with pre-capillary forms of PH, and therefore, it may warn us that this model should be used with caution when evaluating PVR non-invasively in patients with AHF.

### Study limitations

Some limitations should be acknowledged when interpreting the study results. First, this was a single center, non-blinded study. Therefore, a center-specific bias cannot be excluded. Second, CMR non-invasive method was validated with the currently gold standard technique to estimate PVR, RHC. Although, RHC were performed by three expertise interventional cardiologists, several technical inaccuracies can also be reported, including imprecise wedge pressure quantification, or inadequate measurement of cardiac output. Our study population displayed a median PVR value in the range of normality (< 3 UW), representative of HF patients in initial stages of PH-LHD. Finally, the patients here evaluated were all of them hospitalized with acute HF syndromes. Thus, we cannot directly extrapolate these findings to stable phases of the disease.

## Conclusions

In patients with AHF, a non-invasive estimation of PVR using CMR shows lack of accuracy and reliability, as well as a limited capacity to discriminate increased PVR values. Further studies are needed to confirm our findings and continue searching for non-invasive formulas for an accurate estimation of PVR in patients with HF.

## Data Availability

The datasets analysed during the current study are available from ER on reasonable request.
